# Serum HE4 detects recurrent endometrial cancer in patients undergoing routine clinical surveillance

**DOI:** 10.1186/s12885-015-1028-0

**Published:** 2015-02-06

**Authors:** Donal J Brennan, Andreas Hackethal, Kristy P Mann, Irene Mutz-Dehbalaie, Heidi Fiegl, Christian Marth, Andreas Obermair

**Affiliations:** 1 Queensland Centre for Gynaecological Oncology, University of Queensland, School of Medicine, Central Clinical Division, Brisbane, Australia; 2 NHMRC Clinical Trials Centre, University of Sydney, Sydney, NSW Australia; 3 Department of Obstetrics and Gynecology, Innsbruck Medical University, Innsbruck, Austria; 4 Queensland Centre for Gynaecological Oncology, Royal Brisbane and Womens Hospital, Ned Hanlon Building, Herston, QLD 4006 Australia

**Keywords:** Endometrial cancer, HE4, Recurrence

## Abstract

**Background:**

The purpose of this study was to evaluate serum HE4 as a biomarker to detect recurrent disease during follow-up of patients with endometrial adenocarcinoma (EAC).

**Methods:**

We performed a retrospective analysis of 98 EAC patients treated at Innsbruck Medical University, between 1999 and 2009. Twenty-six patients developed recurrent disease. Median follow-up was 5 years. Serum HE4 and CA125 levels were analyzed using the ARCHITECT assay (Abbott, Wiesbaden, Germany) pre-operatively (baseline), post-operative (interval) and after histological confirmation of recurrent disease or when patients returned for clinical review with no evidence of recurrent disease (recurrence/final)). Receiver operator curves (ROC), Spearman rank correlation coefficient, chi-squared and Mann–Whitney tests were used for statistical analysis.

**Results:**

HE4 levels decreased after initial treatment (p = 0.001) and increased again at recurrence (p = 0.002). HE4 was elevated (>70 pmol/L) in 21 of 26 (81%) and CA125 was elevated (>35 U/ml) in 12 of 26 (46%) patients at recurrence. In endometrioid histology (n = 69) serum HE4 measured during follow up (Area under the curve (AUC) = 0.87, 95%CI 0.79-0.95) was a better indicator of recurrence than CA125 (AUC = 0.67, 95%CI 0.52-0.83). A HE4 level of 70 pmol/L was associated with a sensitivity of 84%, a specificity of 74% and a negative predictive value of 93% when assessing for recurrent endometrioid EAC.

**Conclusion:**

This is a preliminary description of HE4 serum levels measured during routine follow up of EAC patients. Serum HE4 measured during clinical follow-up may identify recurrent disease particularly in patients with endometrioid histology. Further prospective validation of HE4 is warranted.

## Background

With more than 300,000 cases occurring annually worldwide, endometrial adenocarcinoma (EAC) is the second most common gynecological cancer [1]. The age-standardized incidence of EAC continues to rise throughout the developed world [1] and this trend is expected to continue mainly due to the increasing prevalence of obesity. While the majority of patients present with early stage disease and thus maintain a reasonable prognosis, 13-17% of women will develop recurrent disease, generally within 3 years of primary treatment [2,3]. Three-year survival following recurrence is ~73% for vaginal recurrence but less than 15% for pelvic or distant recurrence [4]. Moreover, 60% of all recurrences occur in “low risk” patients (endometrioid subtype, low grade and stage) who are not routinely offered adjuvant therapy, and half of these are distant recurrences with a poor prognosis [3].

Traditionally, EAC patients are monitored in follow-up programs for several years after primary treatment. It is expected that recurrence can be detected early and at a time when the tumor volume is smallest implying that treatment of recurrence is effective [3]. Current post-treatment surveillance guidelines differ significantly and schedules are determined mostly by local traditions and personal preferences. A systematic review of 16 retrospective studies on endometrial cancer recurrence found little evidence to support intensive follow-up schedules with regular diagnostic investigations, such as vault cytology, medical imaging or serum tumour markers [2]. Approximately 70% of patients will present with symptomatic recurrence [2,3] and patients with symptomatic recurrence have a worse overall survival than asymptomatic patients [3]. The Society of Gynecologic Oncologists recommend a thorough clinical history, clinical examination and patient education of worrying symptoms as the most effective methods of detecting recurrent EAC and state that at present there is a lack of evidence to support diagnostic interventions such as vault cytology or routine imaging to monitor endometrial cancer patients for recurrent disease [5].

Human epididymis protein 4 (HE4), initially identified as one of four cDNAs highly expressed in the human epididymis [6] is a secreted protein that is overexpressed in patients with serous and endometrioid epithelial ovarian [7,8] and uterine cancers [9-11]. HE4 has proven utility as a serum biomarker in epithelial ovarian cancer (reviewed in [12]) and in 2009, the United States Food and Drug Agency (FDA) approved HE4 as an aid in monitoring recurrence or progressive disease in patients with epithelial ovarian cancer. There is accumulating evidence that HE4 may also prove to be a useful biomarker in EAC. Serum HE4 levels are increased in EAC patients compared to healthy controls [9-11]. Increased HE4 levels are associated with myometrial invasion [9,13-15] and poor prognosis [9,11,13,16], however this study focuses on HE4 and CA125 levels during clinical follow-up after primary treatment.

The aim of this study was a) to describe the kinetics of serum HE4 levels between baseline and the development of recurrent disease and b) to assess the suitability of serial serum HE4 levels as an indicator of recurrence of EAC.

## Methods

### Patients and specimens

Ninety-eight patients with EAC (age 40–85 years, median 65 years), all treated at the Department of Obstetrics and Gynecology, Innsbruck Medical University, between 1999 and 2009 were included in this retrospective study. These patients had been part of a previous study [16], and were selected for this study based on the availability of three consecutive blood samples. Blood was collected at three time points – diagnosis (prior to definitive surgery), interval and final. For the interval time point, blood was collected when patients returned for clinical review and did not have any clinical evidence of recurrent disease. For the final time point, blood was collected after histological confirmation of recurrent disease or when patients returned for another clinical review and did not have any clinical evidence of recurrent disease.

Twenty-six patients developed recurrent disease and 72 remained disease free. Recurrent disease was defined as a histopathologically documented disease after a disease-free interval of 3 or more months. Median follow-up time was 5 years (range 0.6-12.6 years). Clinico-pathological characteristics of patients are outlined in Table 1. Histological classification was performed according to WHO criteria, and stage of disease was determined in accordance with the FIGO guidelines adopted in 1998. Patients with grade 3 tumors, serous papillary or clear cell histology, depth of invasion of more than 50% of myometrium, or stromal infiltration of the cervix were classified as high-risk endometrial cancer. If all factors were negative the patients were classified as low-risk. The extent of surgery depended on disease stage, risk classification and patient operability. Surgery included total hysterectomy and bilateral salpingo-oophorectomy. Para-aortic and pelvic lymph nodes were sampled or completely dissected. Patients with synchronous tumours at diagnosis were excluded from the study. Patient follow-up was performed from the date of primary treatment until last visit or death. For the first three years patients were seen on a three monthly basis including a gynaecological examination and an annual CT-scan. From year 3 to 5 the patients were invited 6 monthly and afterwards yearly. Disease stage, histology, grade, treatment information, age, date of recurrence and date of last follow-up visit or death were recorded in all cases. The study was approved by the Ethics Committee of Innsbruck Medical University (reference number UN4100).

**Table 1:** **Clinicopathological features of 98 patients**

**Age**	
Median (IQR)	65 (59–72)
**Stage**	
FIGO 1	58
FIGO 2	7
FIGO 3	26
FIGO 4	8
**Histology**	
Endometrioid	69
Serous Papillary/Clear cell	22
Carcinosarcoma	8
**Lymph nodes**	
LN negative	76
LN positive	21
Unknown	2
**Grade**	
1	17
2	39
3	42
Unknown	1
**Risk status**	
Low risk	29
High risk	70
**Adjuvant treatment**	
No Adjuvant treatment	5
Radiotherapy	54
Chemotherapy	19
Chemotherapy + radiotherapy	20
**Recurrence**	
None	73
Local	20
Distant	6

*Abreviations*: *IQR* interquartile range, *FIGO* International Federation of Gynecology and Obstetrics, *LN* lymph node.

### Quantitative determination of HE4 and CA125 in human serum

Serum was stored at −80°C until analysis. Specimens were analyzed by means of chemiluminescent microparticle immunoassays specific for CA125 (ARCHITECT CA125 II assay; Abbott GmbH, Wiesbaden, Germany) or for HE4 (ARCHITECT HE4 assay; Abbott GmbH, Wiesbaden, Germany). The dynamic range of HE4 detection goes from 20 to 1500 pM with an automated 1:10 dilution protocol that extends the linear range up to 15,000 pM. The intra-assay and total imprecision (CV%) of the CMIA HE4 assay has previously been demonstrated to range from 2.11 to 2.93 and from 3.13 to 3.70 depending on the concentrations of the positive controls used [17]. The CA125 assay is linear up to 1000 U/mL and has a normality threshold at 35 U/mL.

### Statistical analysis

HE4 and CA125 at each time point were compared using Wilcoxon rank test as data were not normally distributed. All calculations were performed using IBM SPSS Statistics version 20.0.0 (IBM Corporation, Armonk, New York, USA). A p value < 0.05 was considered statistically significant. Receiver operator curves (ROC) were used to compare the ability of HE4 and CA125 to identify patients with recurrent disease. Statistical comparison of ROC curves (StAR) was used to compare AUC values for HE4 and CA125 as previously described [18]. No adjustments have been made for multiple comparisons.

## Results

Ninety-eight patients were identified with blood samples available for the three time points. Median time from diagnosis to interval blood sample was 33 months (range 3–138 months), and median time between interval and final serum samples was 11 months (range 1–122 months). Twenty-six patients developed recurrent disease and 72 patients remained disease free. Thirteen patients (50%) who developed recurrence died of their disease and median survival after recurrence was 14 months (range 0–69 months). Patients who developed recurrent disease were older and were more likely to have stage IV disease at presentation (Table 2). There were no significant differences in other clinic-pathological variables between patients who developed recurrent disease compared to those that remained disease free (Table 2).

**Table 2:** **Clinico-pathological characteristics stratified based on recurrence status**

	No recurrence (n = 73)	Recurrence (n = 26)	P value
**Age**			
Median (IQR)	63 (57–71)	71 (64–77)	0.002^a^
**FIGO Stage**			
1	46 (63)	12 (46)	0.064^b^
2	4 (5)	3 (12)	
3	20 (27)	5 (19)	
4	2 (3)	6 (23)	
**Histology**			
Endometrioid	50 (69)	19 (73)	0.685^b^
Serous Papillary/Clear cell	16 (23)	5 (19)	
Carcinosarcoma	6 (8)	2 (8)	
**Lymph nodes**			
LN negative	58 (80)	18 (72)	0.500^c^
LN positive	13 (20)	7 (28)	
Unknown	1	1	
**Grade**			
1	14 (19)	3 (12)	0.841^b^
2	27 (37)	12 (46)	
3	30 (43)	11 (42)	
Unknown	1		
**Risk status**			
Low risk	18 (25)	11 (42)	0.089^c^
High risk	54 (75)	15 (58)	
**Adjuvant treatment**			
No Adjuvant treatment	5 (6)	2 (8)	0.297^b^
Radiotherapy	44 (60)	10 (39)	
Chemotherapy	10 (14)	9 (35)	
Chemotherapy + radiotherapy	14 (20)	5 (19)	
**Median follow-up**	5.36 (0.6–12.6)	2.63 (0.6–8.6)	
(Years (range))			

Values in parenthesis are percentages unless otherwise stated. IQR – interquartile range, FIGO - International Federation of Gynecology and Obstetrics, LN – lymph node. ^a^Mann–Whitney U test, ^b^Spearman’s Correlation, ^c^Pearson Chi Squared test.

Serum HE4 and CA125 levels were significantly elevated at all 3 time points in the recurrent compared to the disease-free group (Table 3). Focusing initially on the recurrence/final time point 21 of 26 (81%) patients had an elevated HE4 (>70 pmol/L) when recurrent disease was diagnosed. By contrast, only 36% of disease-free patients had an elevated HE4 (>70 pmol/L) during routine post-treatment surveillance (Table 3). Twelve patients (47%) had an elevated CA125 (>35 U/ml) at recurrence. When combined CA125 and/or HE4 were elevated in 23 of 26 (88%) of patients with recurrent disease.

**Table 3:** **CA125 and HE4 levels pre and post treatment for endometrial cancer**

	No reccurence (n = 72)	Recurrence (n = 26)	P value
**Median CA125 (IQR)**			
Diagnosis	39 (20–79)	85 (40–141)	0.001^a^
Interval	11 (8–18)	14 (11–36)	0.033^a^
Final	12 (9–20)	31 (14–90)	<0.001^a^
**Median HE4 (IQR)**			
Diagnosis	67 (47–117)	132 (81–264)	<0.001^a^
Interval	58 (45–89)	82 (64–119)	0.003^a^
Final	57 (43–92)	117 (82–197)	<0.001^a^
**HE4 at Diagnosis**			
< 70 pmol/L	41 (56)	3 (12)	<0.001^b^
>70 pmol/L	32 (44)	23 (88)	
**Interval HE4**			
<70 pmol/L	49 (67)	9 (35)	0.004^c^
>70 pmol/L	24 (33)	17 (65)	
**Final HE4**			
<70 pmol/L	47 (64)	5 (19)	<0.001^c^
>70 pmol/L	26 (36)	21 (81)	
**CA125 at Diagnosis**			
<35 U/ml	35 (48)	4 (15)	0.005^b^
>35 U/ml	38 (52)	22 (85)	
**Interval CA125**			
<35 U/ml	69 (95)	20 (77)	0.019^b^
>35 U/ml	4 (5)	6 (23)	
**Final CA125**			
<35 U/ml	67 (91)	14 (53)	<0.001^c^
>35 U/ml	6 (9)	12 (47)	

Values in parenthesis are percentages unless otherwise stated. IQR – interquartile range. ^a^Mann–Whitney U test, ^b^Fisher’s exact test, ^c^Pearson Chi Squared test.

Examining the five patients with a HE4 less than 70 pmol/L at the time of recurrence (4 local, 1 distant), two patients had an elevated serum CA125 at recurrence and one patient had a HE4 less than 70 pmol/L at diagnosis. Four of these five patients had stage 1 disease (3 endometrioid and 1 papillary serous histology). The fifth patient had stage IV uterine papillary serous carcinoma. All of these patients had full surgical staging and received adjuvant treatment.

**Figure 1:**
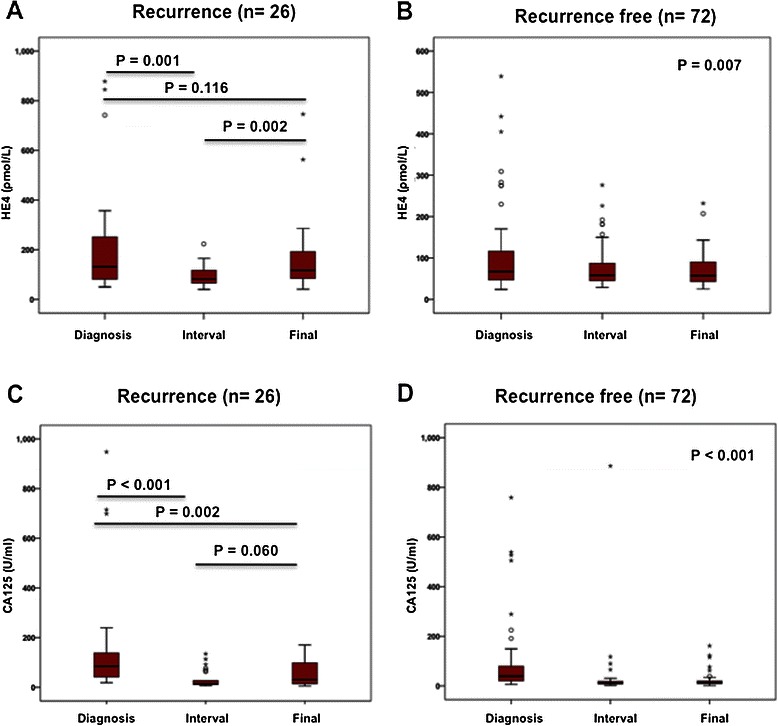
**HE4 and CA125 levels in recurrent endometrial cancer.** Box Plot of HE4 levels at three different time points demonstrating median and interquartile ranges with bars demonstrating 95% confidence intervals for 26 patients who developed recurrent endometrial cancer **(A)** and 72 patients who remained disease free **(B)**. Box Plot of CA125 levels at three different time points demonstrating median and interquartile ranges with bars demonstrating 95% confidence intervals for 26 patients who developed recurrent endometrial cancer **(C)** and 72 patients who remained disease free **(D)**.

Serum HE4 levels decreased after initial treatment (p = 0.001) and increased again at recurrence (p = 0.002) in patients who developed recurrence (Figure 1A). In the 26 patients who developed recurrent disease, serum HE4 levels at diagnosis (median - 132 pmol/L (Inter Quartile Range (IQR) – 81–264 pmol/L)) did not differ significantly from those at recurrence (median −117 pmol/L, IQR 82–197 pmol/L) (p = 0.116). HE4 at baseline did not correlate with HE4 at the interval time point in recurrent patients. In patients who remained disease-free and did not develop recurrence, HE4 levels decreased after diagnosis and remained low for both subsequent time points (Figure 1B).

Serum HE4 levels increased at the interval time point in 3 of 26 patients who subsequently developed recurrent disease, however all of these patients recurred within 3 months of their interval sample, suggesting that they may have had subclinical recurrence at the time of their interval sample. These included two patients with papillary serous carcinoma (stage III and IV) and one patient with stage 2 endometrioid carcinoma. A higher proportion of patients who remained disease free had a HE4 < 70 pmol/L at the interval time point compared to those who subsequently developed recurrent disease (67% vs. 35%, p = 0.004) suggesting that normalization of HE4 after initial treatment may be a prognostic factor (Table 3).

Median serum CA125 levels fell after initial treatment (p < 0.001) and were significantly lower at relapse compared to diagnosis (p = 0.002) (Figure 1C). In disease free patients CA125 levels decreased after diagnosis and remained low for both subsequent time points (Figure 1D). Serum CA125 levels fell in 24 patients after initial treatment. Two patients had an increased CA125 at the interval time-point one of which also had an increased interval HE4 compared to her diagnostic HE4. Both patients had a decrease in their CA125 at recurrence suggesting that their CA125 may have been increased due to other reasons (surgery). CA125 did not increase in a substantial number of patients diagnosed with recurrent disease (Figure 1B, Table 3).

**Figure 2:**
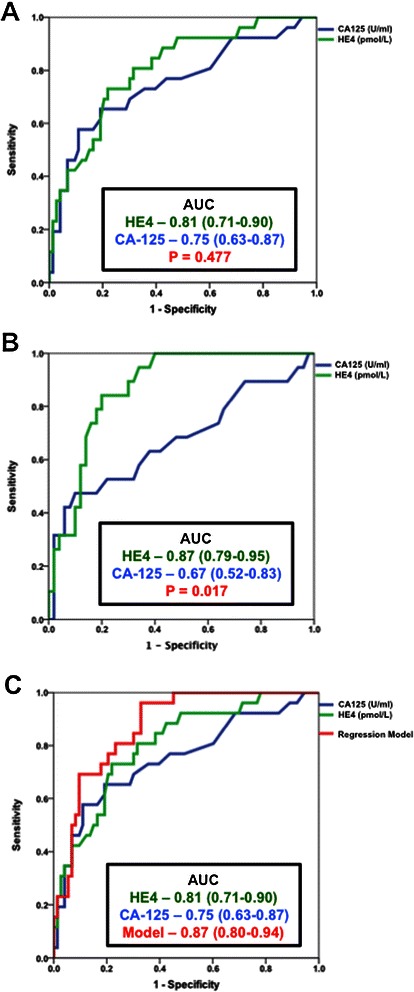
**HE4 is a superior predictor of recurrent disease than CA125.** Receiver operator curves (ROC) comparing HE4 to CA125 for identify patients with recurrent endometrial cancer in all evaluated patients (n = 98) **(A)** and those with endometrioid histology (n = 69) **(B)**. A regression model combining age, CA125 and HE4 did not improve the sensitivity or specificity of HE4 to identify recurrent disease in the entire cohort (n = 98) **(C)**. AUC = area under the curve. Values in parenthesis are 95% confidence interval.

For all patients area-under-the-curve (AUC) analysis of HE4 vs. CA125 measured during follow-up revealed a non-significant increased AUC for HE4 (AUC 0.81; 95% CI 0.71-0.90) compared to CA125 (AUC 0.75; 95% CI 0.63-0.87; p = 0.48) (Figure 2A). The superiority of HE4 (AUC 0.81; 95% CI 0.79-0.95) over CA125 (AUC 0.67; 95% CI 0.52-0.83) (p = 0.017) to indicate recurrence during follow-up was limited to patients with endometrioid cell type (n = 69), (Figure 2B). A logistic regression model combining age, HE4 and CA125 (AUC for model 0.87; 95% CI 0.80-0.94) did not improve the predictive value of HE4 alone (AUC 0.81; 95% CI 0.71-0.90) to indicate recurrence in the entire cohort (Figure 2C) or in the subgroup of patients with endometrioid cell type (data not shown). The sensitivity and specificity of HE4 to indicate recurrence during follow up was assessed using a threshold of 70 pmol/L as previously published [13,15,19]. Serum HE4 levels of 70 pmol/L were associated with a sensitivity of 81%, a specificity of 64% and a negative predictive value of 90% to indicate the presence of recurrence (n = 98) (Table 4). For patients with endometrioid cell type (n = 69), a serum HE4 level of 70 pmol/L was associated with a sensitivity of 84%, a specificity of 74% and a negative predictive value of 93% when assessing for recurrent disease. For all patients a serum CA125 level of 35 U/ml was associated with a sensitivity of 46%, a specificity of 92% and a negative predictive value of 83%. These figures did not change significantly when the analysis was restricted to endometrioid histology, where a serum CA125 level of 35 U/ml was associated with a sensitivity of 42%, a specificity of 92% and a negative predictive value of 81% (Table 4).

**Table 4:** **Sensitivity and specificity of serum HE4 and CA125 measured during post treatment surveillance in detecting recurrent endometrial cancer**

	All patients (n = 98)	
	**HE4 > 70**	**CA125 > 35**
Sensitivity	0.81	0.46
Specificity	0.64	0.92
PPV	0.45	0.67
NPV	0.90	0.83
Overall accuracy	0.69	0.80
	**Endometrioid histology (n = 69)**	
	**HE4 > 70**	**CA125 > 35**
Sensitivity	0.84	0.42
Specificity	0.74	0.92
PPV	0.55	0.67
NPV	0.93	0.81
Overall accuracy	0.77	0.78

PPV – positive predictive value, NPV – negative predictive value.

## Discussion

Long-term surveillance programs for patients treated for primary EAC focus mainly on early detection of recurrent disease, which occurs in 13-17% of women, mostly within 3 years of primary treatment [2,3]. The rationale underlying this approach being that an earlier diagnosis of relapse correlates with a lower volume of disease, more therapeutic options and better outcomes [3]. Consistent with this hypothesis a number of studies have demonstrated that patients with symptomatic recurrence have significantly decreased survival compared to those diagnosed with recurrent disease in an asymptomatic state [3,20]. However, despite intensive surveillance, symptomatic recurrence rates range from 41-83% [5] and there is very little evidence to support the role of routine vaginal cytology, imaging or CA125 in post treatment surveillance of EAC patients [5,20]. It is therefore obvious that a serum biomarker to identify recurrent EAC during post-treatment surveillance would be of immense clinical value.

Herein we present a serial analysis of HE4 as a biomarker to detect recurrent EAC. The only other analysis of its type has only been presented in abstract format [21]. We demonstrate that an elevated HE4 at diagnosis generally falls after initial treatment, but increases again in patients who develop recurrent disease, particularly in patients with endometrioid histology. Furthermore we demonstrate HE4 is elevated in 80% of patients with recurrent EAC and that a HE4 level above 70 pmol/L was associated with a sensitivity of 81%, a specificity of 64% and a negative predictive value of 90% when assessing for recurrent disease. We also show that HE4 is particularly relevant in patients with endometriod histology. A 70 pmol/L threshold for HE4 was chosen to identify recurrent disease as this threshold has previously been used by us and others to identify deeply invasive tumours [13,15] and was recently identified in a critical review as the most sensitive and specific threshold for primary EAC diagnostic studies [19]. The addition of CA125 did not improve the sensitivity or specificity of HE4 to detect recurrent EAC. The sensitivity values reported herein for HE4 are significantly higher than those reported in historical studies of CA125 in recurrent EAC [22-24].

The strengths of this study include the fact that this is one of the first studies of HE4 in recurrent EAC. We used a relatively large cohort and all patients were fully surgically staged. Serum samples were managed in standardized fashion. Weaknesses of the study include the fact that this was a single institutional study and samples were simply selected based on the availability of three serum samples. In addition there were a relatively small proportion of distant recurrences. We also only had access to samples at three time points and thus were not able to assess dynamic changes in HE4 and CA125 as predictors of recurrence. Although renal failure is a well-recognized cause of elevated HE4 in benign disease, we were unable to control for this as baseline renal function was not available for patients in this study. Finally we did not have data on whether patients presented with symptomatic or asymptomatic recurrence, which would be helpful when determining the clinical relevance of HE4 in detecting recurrent EAC.

These data suggest that HE4 may be an effective biomarker in post treatment surveillance of EAC. The dynamics of HE4 in EAC are an elevation in a significant proportion of patients at diagnosis, a significant decrease after initial surgery followed by another significant rise if recurrent disease develops. In contrast we and others have previously demonstrated that patients with a low pre-operative HE4 generally have early stage disease with a low risk of developing recurrence and may not require intensive post operative surveillance [13,16]. Although these findings are preliminary and require validation in independent prospective cohorts, they suggest that HE4 may be used to triage and monitor EAC patients at high risk of recurrence, particularly in patients with low-grade endometrioid histology who would generally be considered at low risk of developing recurrent disease. Further studies are required to validate the 70 pmol/L threshold and also to assess the correct time intervals for HE4 analysis in the follow-up schedule. In addition further longitudinal studies are required to investigate the importance of dynamic changes in HE4 over time in a similar fashion to how PSA is used in prostate cancer and CA125 in ovarian cancer screening studies.

## Conclusion

In summary, these data are a preliminary description of HE4 in recurrent EAC and suggest that it may be a sensitive and specific predictor of recurrent disease particularly in patients with endometrioid histology. HE4 is elevated in 81% of patients with recurrent endometrioid EAC and is significantly superior than CA125 as a predictor of recurrent disease. The sensitivity and NPV values presented are similar to those published for CA125 in post treatment surveillance of epithelial ovarian cancer [5,25] suggesting that further prospective analysis of HE4 in post treatment surveillance of EAC is warranted. Given the fact that following CA125 in post treatment surveillance of ovarian cancer does not impact on survival [26], future studies of HE4 in endometrial cancer follow-up should focus on the impact of HE4 on clinical decision-making and whether it has any impact on survival.
